# An Efficient ISAR Imaging of Targets with Complex Motions Based on a Quasi-Time-Frequency Analysis Bilinear Coherent Algorithm

**DOI:** 10.3390/s18092814

**Published:** 2018-08-26

**Authors:** Cao Zeng, Mengyi Qin, Dong Li, Hongqing Liu, Yi Chai

**Affiliations:** 1 National Laboratory of Radar Signal Processing, Xidian University, Xi’an 710071, China; czeng@mail.xidian.edu.cn; 2 Center of Communication and Tracking Telemetering Command, Chongqing University, Chongqing 400044, China; cqumyqin@163.com; 3 Key Laboratory of Complex System Safety and Control, Ministry of Education, Chongqing University, Chongqing 400044, China; chaiyi@cqu.edu.cn; 4 Guangxi Key Laboratory of Wireless Wideband Communication and Signal Processing, Guilin University of Electronic Technology, Guilin 541004, China; 5 Chongqing Key Laboratory of Mobile Communications Technology, Chongqing University of Posts and Telecommunications, Chongqing 400065, China; hongqingliu@outlook.com

**Keywords:** inverse synthetic aperture radar (ISAR), maneuvering targets, radon-CPF-Fourier transform (RCFT), low SNR environment

## Abstract

The inverse synthetic aperture radar (ISAR) imaging for targets with complex motions has always been a challenging task due to the time-varying Doppler parameter, especially at the low signal-to-noise ratio (SNR) condition. In this paper, an efficient ISAR imaging algorithm for maneuvering targets based on a noise-resistance bilinear coherent integration is developed without the parameter estimation. First, the received signals of the ISAR in a range bin are modelled as a multicomponent quadratic frequency-modulated (QFM) signal after the translational motion compensation. Second, a novel quasi-time-frequency representation noise-resistance bilinear Radon-cubic phase function (CPF)-Fourier transform (RCFT) is proposed, which is based on the coherent integration of the energy of auto-terms along the slope line trajectory. In doing so, the RCFT also effectively suppresses the cross-terms and spurious peaks interference at no expense of the time-frequency resolution loss. Third, the cross-range positions of target’s scatters in ISAR image are obtained via a simple maximization projection from the RCFT result to the Doppler centroid axis, and the final high-resolution ISAR image is thus produced by regrouping all the range-Doppler frequency centroids. Compared with the existing time-frequency analysis-based and parameter estimation-based ISAR imaging algorithms, the proposed method presents the following features: (1) Better cross-term interference suppression at no time-frequency resolution loss; (2) computationally efficient without estimating the parameters of each scatters; (3) higher signal processing gain because of 2-D coherent integration realization and its bilinear function feature. The simulation results are provided to demonstrate the performance of the proposed method.

## 1. Introduction

Thanks to the ability to produce high-resolution microwave imagery for the non-cooperative target nearly regardless of weather condition, the inverse synthetic aperture radar (ISAR) presents a range of applications in the field of national defense surveillance [1,2,3,4]. The range-Doppler (RD) ISAR imaging algorithm [5,6] can be an effective means to obtain radar images, provided that the Doppler frequency shift is constant during the imaging time. However, in the case of non-cooperative and/or high maneuvering targets, the received signal in a range bin is usually complex, and studies show that a multicomponent polynomial phase signal (PPS) is an accurate model. Therefore, the Fourier transform (FT) based RD approach cannot handle this model well and the Doppler spectrum produced is spread out, and the radar images obtained by those methods are blurry [6].

Recently, a new ISAR strategy termed Range Instantaneous Doppler (RID) algorithm was proposed to produce ISAR image of a maneuvering target [7,8,9,10,11,12,13,14,15,16]. The RID algorithms are usually divided into two groups. The first group is the parametric approach, where the received signal in a range bin is modeled by a special signal [7,8,9], and based on that, the instantaneous Doppler frequency is estimated and the corresponding ISAR image is obtained by using the estimated Doppler frequency. However, those approaches require the estimation or extraction of each scatterer in each range cell, which is computationally demanding and inefficient. In addition, model mismatch issue is usually present, resulting in unfocused images [9]. The second group is the nonparametric method [10,11,12,13,14,15,16], in which time-frequency distribution (TFD) analysis tool as the substitution of FT in the azimuth focusing processing, is utilized. The TFD analysis overcomes the model mismatch issue and can be implemented efficiently, and therefore in this paper the TFD is also considered. The TFD based imaging methods such as the short-time Fourier transform (STFT) [10], continuous wavelet transform (CWT) [11], were widely explored. The STFT and CWT are free from the cross-term interference, but the resolution is low. The Wigner-Ville distribution (WVD) method [12] provides high-resolution time-frequency analysis. However, its performance deteriorates when the cross-term interferences are severe for processing multicomponent PPS. To reduce the cross-term interferences, the smoothed pseudo Wigner-Ville distribution (SPWVD) algorithm [13] and the L-class of fourth-order complex-lag PWVD algorithm [14] were proposed. However, a compromise must be made between the capacity to suppress cross-term interference and the time-frequency resolution. It is known that maximizing cross term suppression without the time-frequency resolution loss is still a major challenge faced by the TFD analysis community. In [15,16], an efficient range centroid Doppler (RCD) ISAR imaging algorithm based a new quasi-time-frequency transform named Lv’s distribution was proposed, where the cross-terms suppression is better achieved with no time-frequency resolution loss. However, this method is only valid for linear frequency modulated (LFM) signals, and its performance degrades dramatically in the case of quadratic frequency-modulated (QFM) signal that often is utilized to model the maneuvering targets. Therefore, the method for maneuvering targets imaging still needs further investigations.

In this work, after the translational motion compensation, the received signal in a range bin is modelled as multi-component PPS signals, and then an efficient ISAR imaging method without estimating parameters for the maneuvering target based on a novel noise-resistance bilinear Radon-cubic phase function (CPF)-Fourier transform (RCFT) is proposed. The RCFT aims at performing the coherent integration of the energy of auto-terms along the slope line trajectory in the time-frequency distribution plane. In the proposed RCFT, the signal-to-noise ratio (SNR) is enhanced and the troublesome cross-terms and spurious peaks interference are effectively suppressed. Finally, the cross-range positions of target’s scatters in ISAR image are obtained via a simple maximization projection from the RCFT result to the Doppler centroid axis, and the high-resolution ISAR image is thus produced by regrouping all the range-Doppler frequency centroids. Compared with the time-frequency analysis based ISAR imaging approaches, the proposed RCFT replaces the FT in the traditional RD algorithm. The main advantages are: (1) the cross-term interferences suppression is realized at no time-frequency resolution loss; (2) a higher signal processing gain is obtained because of 2-D coherent integration realization and its bilinear function feature. Compared with the parameter estimation based ISAR imaging approaches, the proposed RCFT enjoys computationally efficiency since parameter estimation of each scatter is not required.

The rest of the paper is organized as follows. In Section 2, the characteristic of the received signal for the maneuvering target is discussed. In Section 3, the RCFT derivation and the ISAR imaging algorithm of the maneuvering target based on RCFT are presented. The experimental results and computational complexity analysis are given in Section 4. Section 5 is the conclusion of this paper.

## 2. ISAR Imaging Model of Maneuvering Target

**Figure 1:**
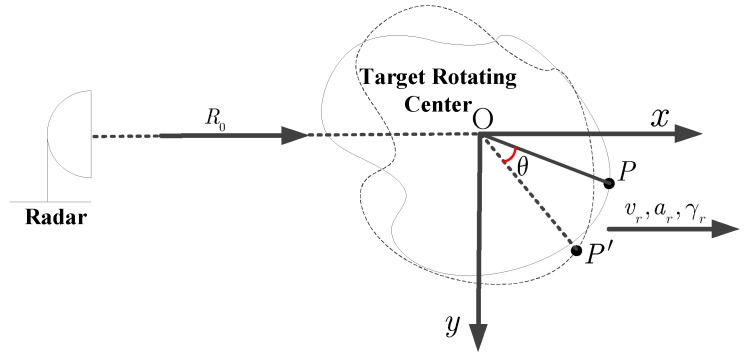
Inverse synthetic aperture radar (ISAR) imaging geometric model of a maneuvering target.

In this section, we do not consider the motion compensation problem, which means that the standard range alignment and phase adjustment are implemented beforehand. In Figure 1, the ISAR geometric configuration of a target with the complex motion is depicted, where XOY is a Cartesian coordinate and the origin O is the position of target rotating center. In Figure 1, the $R_{0}$ denotes the initial distance of origin O to radar platform, and $v_{r}$,$\;a_{r}$, and $\gamma_{r}$ respectively represent the radial velocity, the acceleration, and the acceleration rate. With those notations, the instantaneous rotation angle $\theta \left(t_{m}\right)$ is

(1) $$\;\theta \left(t_{m}\;\right)=\;\int_{0}^{t_{m}}\omega \left(t_{m}\right){\mathrm{dt}}_{m}\;\approx {\;\theta }_{0}{\;+\;\Omega t}_{m}\;+\;\frac{1}{2}\Omega^{\prime }t_{m\;}^{2}+\;\frac{1}{6}\Omega^{\prime \prime }t_{m}^{3}$$

where $t_{m}$ indicates the slow time variable,$\;\omega \left(t_{m}\right)$ is the rotation angular velocity at time $t_{m}$, and Ω, $\Omega^{\prime }$ and $\Omega^{\prime \prime }$ respectively denote the initial angular velocity, angular acceleration, and angular acceleration rate.

From (1), $R_{s}\left(t_{m}\right)$ is the slant range of the scatter *P* with position $\left(x_{p},y_{p}\right)$ at time $t_{m}$, given by

(2) $$\;R_{s}\left(t_{m}\;\right)\approx R_{0}+v_{r}t_{m}+\frac{1}{2}a_{r}t_{m}^{2}+\frac{1}{6}\gamma_{r}t_{m}^{3}+x_{p}cos\;\theta \left(t_{m}\right)-y_{p}sin\;\theta \left(t_{m}\right)$$

In practice, the coherent integration time is usually short, and therefore the target rotation angle during that time is small, i.e., $3–5^{\circ }$ [8,9]. From that, $sin\;\theta \left(t_{m}\right)$ and $cos\;\theta \left(t_{m}\right)$ are approximated by $\theta \left(t_{m}\right)$ and 1. With also the assumption that motion compensation is completed, the received azimuth signal in a range bin is

(3) $$\begin{array}{l} \;s_{r}\left(t_{m}\;\right)\;\approx \overset{K}{\underset{k=1}{\sum }}A_{k}\exp\left[j\frac{4\pi f_{c}}{c}\left(R_{0}+x_{k}+\left(v_{r}-y_{k}\Omega \right)t_{m}+\frac{1}{2}\left(a_{r}-{\;y}_{k}\Omega^{\prime }\right)t_{m}^{2}+\frac{1}{6}\left(\gamma_{r}-y_{k}\Omega^{\prime \prime }\right)t_{m}^{3}\right)\right]\\ \;+n\left(t_{m}\right) \end{array}$$

where $f_{c}$, c, $A_{k}$ and K respectively represent the carrier frequency, the velocity of the wave propagation, the magnitude of the kth point scatterer, and the number of point scatterers in one range cell, $n\left(t_{m}\right)$ is additive complex white Gaussian noise with a variance of $\delta^{2}$. From (3), it is seen that the received signal in a range cell is a multi-component PPS. In a generic form, (3) is rewritten as

(4) $$\;\;s_{r}\left(t_{m}\right)\approx \overset{K}{\underset{k=1}{\sum }}A_{k}\exp\left[j2\pi \phi_{k}\left(t_{m}\right)\right]+n\left(t_{m}\right)\;=\overset{K}{\underset{k=1}{\sum }}A_{k}\exp\left(j2\pi \left(b_{k,0}+b_{k,1}t_{m}+b_{k,2}t_{m}^{2}++b_{k,3}t_{m}^{3}\right)\right)+n\left(t_{m}\right)\;$$

where $\phi_{k}\left(t_{m}\right)$ is the phase term, and $b_{k,0}$, $b_{k,1}$,$\;b_{k,2}$ and $b_{k,3}$ respectively represent the initial phase, the centroid frequency, the chirp rate, and the change rate of chirp rate. From (4) again, it is clearer that the azimuth signal in a range bin is a multicomponent QFM signal. In this case, the conventional FT is not suitable to process QFM signal, and the second and high-order terms in (4) will deteriorate the image if they are not properly handled. Therefore, in this work, a novel RCFT is developed as a substitution of FT in the azimuth focusing.

## 3. Proposed Algorithm Description

For radar imaging of non-uniformly rotating target, the radar echo signals in a range cell can be characterized as multicomponent QFM signals after migration compensation, which has been illustrated in Section 2. In this section, an efficient ISAR imaging of targets with complex motions based on a noise-resistance bilinear coherent RCFT is proposed.

### 3.1. Description of the Proposed RCFT

In the CPF approach proposed in [17,18], instantaneous autocorrelation function of (4) is defined as

(5) $$\begin{array}{l} \;R_{s}^{C}\left(t_{m};\tau_{m}\;\right)=s_{r}\left(t_{m}+\tau_{m}\right)s_{r}\left(t_{m}-\tau_{m}\right)\\ \;=\underset{\mathrm{auto}-\mathrm{terms}}{\underbrace{\overset{K}{\underset{k=1\;}{\sum }}A_{k}^{2}\exp\left[j4\pi \phi_{k}\left(t_{m}\right)+j2\pi \left(b_{k,2}+3b_{k,3}t_{m}\right)\tau_{m}^{2}\right]+\underset{\mathrm{cross}-\mathrm{terms}}{\underbrace{R_{s,c-terms}\left(t_{m};\tau_{m}\right)}}}}\\ \;+\underset{\mathrm{noise}-\mathrm{terms}}{\underbrace{R_{s,n-terms\;}\left(t_{m};\tau_{m}\right)}} \end{array}$$

where $\tau_{m}$ is the lag time variable. The $R_{s,c-terms}\left(t_{m};\tau_{m}\right)$ and $R_{s,n-terms}\left(t_{m};\tau_{m}\right)$ are the cross-terms and the noise terms, respectively, and detailed expressions can be found in [17].

Taking the Fourier transform (FT) to (5) along the lag time variable $\tau_{m}$, one can obtain the CPF

(6) $$\begin{array}{c} \;\mathrm{CPF}\left(t_{m},f_{\tau_{m}^{2}\;}\right)=\int R_{s}^{C}\left(t_{m};\tau_{m}\right)\exp\left(-j2\pi f_{\tau_{m}^{2}}\tau_{m}^{2}\right)d\tau_{m}^{2}\\ =\underset{\mathrm{auto}-\mathrm{terms}}{\underbrace{\overset{K}{\underset{k=1}{\sum }}A_{k}^{2}\exp\left[j4\pi \phi_{k}\left(t_{m}\right)\right]\delta_{\tau_{m}^{2}}\left[f_{\tau_{m}^{2}}-\left(b_{k,2}+3b_{k,3}t_{m}\right)\right]}}+\underset{\mathrm{cross}-\mathrm{terms}}{\underbrace{{\mathrm{CPF}}_{s,c-terms}\left(t_{m};f_{\tau_{m}^{2}}\right)}}\\ +\underset{\mathrm{noise}-\mathrm{terms}}{\underbrace{{\mathrm{CPF}}_{s,n-terms}\left(t_{m};f_{\tau_{m}^{2}}\right)}} \end{array}$$

where $f_{\tau_{m}^{2}}$ is the frequency variable corresponding to the lag variable $\tau_{m}$ and δ(·) is the Dirac delta function. The ${\mathrm{CPF}}_{s,c-terms}\left(t_{m};f_{\tau_{m}^{2}}\right)$ and ${\mathrm{CPF}}_{s,n-terms}\left(t_{m};f_{\tau_{m}^{2}}\right)$ are the cross term and the noise term after the FT.

Note also that from (6), the CPF performs the discrete FT (DFT) in terms of the lag time variable $\tau_{m}^{2}$ whose sampling grid is non-uniform. As a result, the traditional uniform-sampling based efficient fast FT (FFT) cannot be directly applied. To calculate the CPF, the non-uniform discrete Fourier transform (NUDFT) is usually utilized, and the cost of the direct usage of NUDFT is as high as $O\left(N_{\tau_{m}}^{2}\right)$ with the signal length $N_{\tau_{m}}$. To reduce the computational cost of the NUDFT, the non-uniform FFT (NUFFT) [19] is preferred, and its computational cost of performing the FT along the $\tau_{m}$ axis is $O\left(2N_{\tau_{m}}\log_{2}N_{\tau_{m}}\right)$ without the performance loss. For more information, the detailed implementation procedures on NUFFT can be found in [19].

Applying the NUFFT in (5) produces the CPF that is the same as in (6) as

(7) $$\begin{array}{l} \;\text{CPF}\left(t_{m},f_{\tau_{m}^{2}}\right)={\text{NUFFT}}_{\tau_{m}^{2}}\left[R_{s}^{C}\left(t_{m};\tau_{m}\right)\right]\\ =\underset{\mathrm{auto}-\mathrm{terms}}{\underbrace{\overset{K}{\underset{k=1\;}{\sum }}A_{k}^{2}\exp\left[j4\pi \phi_{k}\left(t_{m}\right)\right]\delta_{\tau_{m}^{2}}\left[f_{\tau_{m}^{2}}-\left(b_{k,2}+3b_{k,3}t_{m}\right)\right]}}\\ +\underset{\mathrm{cross}-\mathrm{terms}}{\underbrace{{\mathrm{CPF}}_{s,c-terms}\left(t_{m};f_{\tau_{m}^{2}}\right)}}+\underset{\mathrm{noise}-\mathrm{terms}}{\underbrace{{\mathrm{CPF}}_{s,n-terms}\left(t_{m};f_{\tau_{m}^{2}}\right)}} \end{array}$$

where ${\mathrm{NUFFT}}_{\tau_{m}^{2}}$ is NUFFT operator along the lag time variable $\tau_{m}^{2}$. 

**Figure 2:**
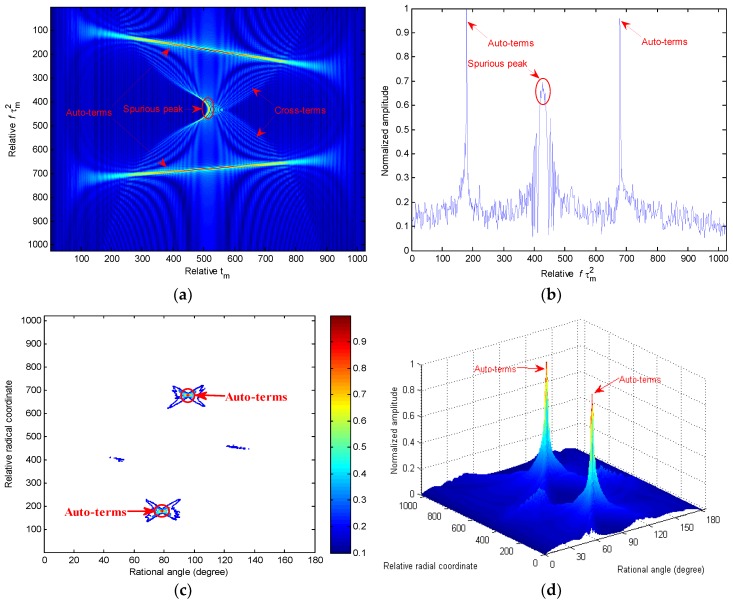
Simulation results of the multi-component QFM signal. (**a**) The results in the $t_{m}-f_{\tau_{m}^{2}}$ plane after CPF operation; (**b**) the slice obtained by the CPF at $t_{m}=512$; (**c**) Contour image of QFM signal obtained by the proposed RCFT method; (**d**) mesh image of QFM signal obtained by the proposed RCFT method.

In (6), because of the nonlinear coupling between azimuth slow-time variable $t_{m}$ and the lag time variable $\tau_{m}^{2}$, the energy of the auto-terms concentrates along the line of $f_{\tau_{m}^{2}}=b_{k,2}+3b_{k,3}t_{m}$ in the slow time-Doppler frequency $t_{m}-f_{\tau_{m}^{2}}$ plane. Utilizing this line, the third- and second-order coefficients are extracted by the slope and the y-intercept [17]. However, in the case of multi-component QFM signal, the identifiability of the CPF is of problem because of cross-terms and spurious peaks [17,18]. To visually demonstrate this issue, the CPF of a two-component QFM signal with length N=2024 with parameters $\sigma_{1}=\sigma_{2}=1$, $b_{1,1}=-0.1$, $b_{1,2}=5\times 10^{-4}$ and $b_{1,3}=-1\times 10^{-7}$ $b_{2,1}=0.1$, $b_{2,2}=-1\times 10^{-3}$, and $b_{2,3}=2\times 10^{-7}$ is shown in Figure 2. From Figure 2a,b, a sharp spurious peak is of presence, and the energy of auto-terms is focused along the slope lines (i.e., $f_{\tau_{m}^{2}}=b_{k,2}+3b_{k,3}t_{m},k=1,2$), whereas the energy of cross-terms is diffused in the $\left(t_{m},f_{\tau_{m}^{2}}\right)$ domain since their positions vary with time $t_{m}$. Generally speaking, for a *K*-component QFM signal, there are $K^{2}-K$ cross-terms and $\left(K^{2}-K\right)/2$ spurious peaks [17], which pose a serious interference to the estimation and detection of the auto-terms, unless they are properly reduced.

To overcome the identifiability problem of the CPF, the cross-terms and spurious peaks must be properly reduced. To this goal, two approaches of the Radon-CPF transform (RCT) in [18] and Hough generalized high-order ambiguous function (Hough-GHAF) method in [20] have been developed to suppress the cross-terms, spurious peaks for multicomponent QFM signal. To utilize the energy of auto-terms, for the RCT, the integration was performed along the slope line defined by the polar distance $\rho_{T}$ (radius) from the origin and polar angle $\theta_{T}$ formed by the perpendicular to the line in the radon domain. The RCT method $S_{\mathrm{RCT}}\left(\rho_{T};\theta_{T}\right)$ is given by [16]

(8) $$\begin{array}{c} S_{\mathrm{RCT}\;}\left(\rho_{T};\theta_{T}\right)=\Psi_{t_{m}}\left[\mathrm{CPF}\left(t_{m},f_{\tau_{m}^{2}}\right)\right]\\ =\int_{-T_{a}/2}^{+T_{a}/2}\left|\mathrm{CPF}\left(t_{m},f_{\tau_{m}^{2}}\right)\right|\delta \left[\rho_{T}-t_{m}\cos\theta_{T}-f_{\tau_{m}^{2}}\text{sin }\theta_{T}\right]dt_{m} \end{array}$$

where $\Psi_{t_{m}}$ represents the RCT operator on $\mathrm{CPF}\left(t_{m},f_{\tau_{m}^{2}}\right)$. From (8), the integral accumulates all the energy of auto-terms and suppresses the cross-terms and spurious peaks.

The similar idea based on multilinear function of fourth-order GHAF is also adopted by Hough-GHAF method in [20]. The Hough-GHAF is defined by

(9) $$\begin{array}{l} \;\;\;S_{\mathrm{Hough}-\mathrm{GHAF}\;}\left(\rho_{T};\theta_{T}\right)=\Theta_{t_{m}}\left[\mathrm{GHAF}\left(t_{m},f_{\tau_{m}}\right)\right]\\ \;=\int_{-T_{a}/2\;}^{+T_{a}/2}\left|\mathrm{GHAF}\left(t_{m},f_{\tau_{m}}\right)\right|\delta \left[\rho_{T}-t_{m}\cos\theta_{T}-f_{\tau_{m}^{2}}\sin\;\theta_{T}\right]dt_{m} \end{array}$$

where $\Theta_{t_{m}}$ represents the Hough-GHAF transform operator. The detailed definition of the $\mathrm{GHAF}\left(t_{m},f_{\tau_{m}}\right)$ can be found in [18]. It is worth mentioning that, the Hough-GHAF is based on a multilinear function of fourth-order, and thus its SNR threshold is higher than RCT. From both (8) and (9), although the energy of auto-terms is explored, the operations in RCT and Hough-GHAF are not coherent, and therefore, the suppression ability of the cross-terms and noise is still not adequate.

To perform coherent integration, the RCFT is developed that fully exploits the energy of the auto-terms. However, the difficulty of performing coherent integration comes from the fact that the cubic and quadratic power terms of $t_{m}$ are present in auto-terms of (6). They are must be eliminated first because the peaks of auto-terms would be unfocused if the direct integration along $t_{m}$ were utilized. In what follows, a novel RCFT algorithm is proposed to coherently integrate the energy of auto-terms along slope lines.

First, to eliminate the effect of the quadratic power terms of $t_{m}$ in (6), here, we intelligently exploit the idea of the sampling property of the Dirac delta function, which is

(10) $$\;\delta \left(t_{m}-t_{c}\;\right)g\left(t_{m}\right)=g\left(t_{c}\right)\delta \left(t_{m}-t_{c}\right)$$

where $g\left(t_{m}\right)$ is a general function of the variable $t_{m}$, and $t_{c}$ denotes a fixed time index.

According to (10), in order to utilize sampling property of the Dirac delta function, an appropriate phase term function should be designed to construct a same expression with the delta function. With this thinking, a modified CPF (MCPF) by utilizing the sampling property of the delta function is designed as

(11) $$\begin{array}{l} \;\mathrm{MCPF}\left(t_{m},f_{\tau_{m}^{2}\;}\right)=\mathrm{CPF}\left(t_{m},f_{\tau_{m}^{2}}\right)\exp\left[-j4\pi f_{\tau_{m}^{2}}t_{m}^{2}\right]\\ =\underset{\mathrm{auto}-\mathrm{terms}}{\underbrace{\overset{K}{\underset{k=1}{\sum }}A_{k}^{2}\exp\left[j4\pi \left(f_{\tau_{m}^{2}}-\left(b_{k,2}+3b_{k,3}t_{m}\right)t_{m}^{2}\right)\right]\delta_{\tau_{m}^{2}}\left[f_{\tau_{m}^{2}}-\left(b_{k,2}+3b_{k,3}t_{m}\right)\right]}}\\ \;\times \underset{\mathrm{auto}-\mathrm{terms}}{\underbrace{\exp\left[j4\pi b_{k,1}t_{m}-j8\pi b_{k,3}t_{m}^{3}\right]}}+\underset{\mathrm{cross}-\mathrm{terms}}{\underbrace{{\mathrm{MCPF}}_{cterms}\left(t_{m};f_{\tau_{m}^{2}}\right)}}\\ =\underset{\mathrm{auto}-\mathrm{terms}}{\underbrace{\overset{K}{\underset{k=1\;}{\sum }}A_{k}^{2}g\left(f_{\tau_{m}^{2}}=b_{k,2}+3b_{k,3}t_{m}\right)\delta_{\tau_{m}^{2}}\left[f_{\tau_{m}^{2}}-\left(b_{k,2}+3b_{k,3}t_{m}\right)\right]}}\times \underset{\mathrm{auto}-\mathrm{terms}}{\underbrace{\exp\left[j4\pi b_{k,1}t_{m}-j8\pi b_{k,3}t_{m}^{3}\right]}}\\ \;+\underset{\mathrm{cross}-\mathrm{terms}}{\underbrace{{\mathrm{MCPF}}_{cterms}\left(t_{m};f_{\tau_{m}^{2}}\right)}} \end{array}$$

where $g\left(t_{m},f_{\tau_{m}^{2}}\right)=\exp\left[j4\pi \left(f_{\tau_{m}^{2}}-\left(b_{k,2}+3b_{k,3}t_{m}\right)t_{m}^{2}\right)\right]$. It is now clear from (11) that the negative effects of the quadratic power of $t_{m}$ in auto-terms are removed by means of the sampling property of the Dirac delta function. This is the first important step in the proposed RCFT algorithm.

It is also found from (11) that the cubic power term of $t_{m}$ just corresponds to the slope of the auto-terms energy distribution in the time-frequency plane. Therefore, to eliminate the effect of the cubic power terms of $t_{m}$ in (5), inspired by the Radon-Fourier-transform (RFT) in [21,22], a novel RCFT algorithm is defined by

(12) $$\begin{array}{l} S_{\mathrm{RCFT}\;}\left(f_{t_{m}};\rho_{T};\theta_{T}\right)=\Gamma_{t_{m}}\left[\mathrm{MCPF}\left(t_{m},f_{\tau_{m}^{2}}\right)\right]\\ =\int_{-T_{a}/2\;}^{+T_{a}/2}\mathrm{MCPF}\left(t_{m},f_{\tau_{m}^{2}}\right)H_{\mathrm{Kernel}}\left(t_{m},f_{\tau_{m}^{2}}\right)\delta \left[\rho_{T}-t_{m}\cos\;\theta_{T}-f_{\tau_{m}^{2}}\sin\theta_{T}\right]dt_{m} \end{array}$$

where $\Gamma_{t_{m}}$ denotes the RCFT operator. The $H_{\mathrm{Kernel}}\left(t_{m},f_{\tau_{m}^{2}}\right)$ in (12) is a novel transform kernel function that is given by

(13) $$\;H_{\mathrm{Kernel}\;}\left(t_{m},f_{\tau_{m}^{2}}\right)=\exp\left[j8\pi \frac{\tan\left(\theta_{T}\right)}{3}t_{m}^{3}-j2\pi f_{t_{m}}t_{m}\right]$$

where $f_{t_{m}}$ is the frequency variable with respect to $t_{m}$. Note that in the case of zero cubic term, i.e., $b_{3}=0$, the proposed RCFT reduces to the coherent integrated CPF (CICPF) approach proposed in our previous work [9]. Moreover, when both the second- and three-order terms are zeros, i.e., $b_{3}=b_{2}=0$, the proposed RCFT becomes the FT, which indicates that FT is a special case of the RCFT.

Substituting (11) into (12) and after reassigning yields

(14) $$\begin{array}{l} S_{\mathrm{RCFT}\;}=\underset{\mathrm{auto}-\mathrm{terms}}{\underbrace{\overset{K}{\underset{k=1}{\sum }}\sigma_{k}^{2}\int \delta_{\tau_{m}^{2}}\left[f_{\tau_{m}^{2}}-\left(b_{k,2}+3b_{k,3}t_{m}\right)\right]\exp\left[-j2\pi \left(f_{t_{m}}-2b_{k,1}\right)t_{m}\right]}}\\ \;\times \underset{\mathrm{cross}-\mathrm{terms}}{\underbrace{\delta \left[\rho_{T}-t_{m}\cos\theta_{T}-f_{\tau_{m}^{2}}\sin\theta_{T}\right]\exp\left[j8\pi \left(b_{k,3}-\tan\left(\theta_{T}\right)/3\right)t_{m}^{3}\right]dt_{m}}}\\ \;+\underset{\mathrm{cross}-\mathrm{terms}}{\underbrace{{\mathrm{RCFT}}_{cterms}\left(f_{t_{m}};\rho_{T};\theta_{T}\right)}} \end{array}$$

where $\sigma_{k}^{2}=A_{k}^{2}g\left(f_{\tau_{m}^{2}}=b_{k,2}+3b_{k,3}t_{m}\right)$. In (14), when the slope of the searching slope line $\delta \left[\rho_{T}-t_{m}\cos\theta_{T}-f_{\tau_{m}^{2}}\sin\theta_{T}\right]$ matches the slope of the auto-terms energy distribution $\delta_{\tau_{m}^{2}}\left[f_{\tau_{m}^{2}}-\left(b_{k,2}+3b_{k,3}t_{m}\right)\right]$, namely $\tan\left(\theta_{T}\right)=3b_{k,3}$, the cubic power of $t_{m}$ in auto-terms is eliminated, also demonstrated by RFT method [21]. Therefore, the proposed RCFT in (14) is capable of realizing the coherent integration for auto-terms while suppressing the cross-terms and spurious peaks. This is the second important step in the proposed RCFT algorithm. Moreover, when the searching slope line fully overlaps with the energy distribution slope line of the auto-terms, namely $\tan\left(\theta_{T}\right)=3b_{k,3}$ and $\rho_{T}=b_{k,2}\cos\;\theta_{T}$, the proposed RCFT maximizes the output energy of auto-terms and produces a distinct peak in which the maximal output energy $E_{\max}$ is calculated by

(15) $$\begin{array}{ll} E_{\max} & ={\Gamma_{t_{m}}\left[\mathrm{MCPF}\left(t_{m},f_{\tau_{m}^{2}}\right)\right]|}_{\rho_{T}=b_{k,2}\cos\;\theta_{T};\theta_{T}=\tan^{-1}\left(3b_{k,3}\right)}\\ & =G_{\mathrm{FT}\;}\sigma_{k}^{2}\delta_{t_{m}}\left(f_{t_{m}}-2b_{k,1}\right)\delta \left[\rho_{T}-t_{m}\cos\;\theta_{T}-f_{\tau_{m}^{2}}\sin\;\theta_{T}\right]\\ & +{{\mathrm{RCFT}}_{cterms}\left(f_{t_{m}};f_{\tau_{m}^{2}}\right)|}_{\rho_{T}=b_{k,2}\cos\;\theta_{T};\theta_{T}=\tan^{-1}\left(3b_{k,3}\right)} \end{array}$$

where $G_{\mathrm{FT}}$ is the FT coherent integration gain. Meanwhile, when $\tan\left(\theta_{T}\right)\neq 3b_{k,3}$ or $\rho_{T}\neq b_{k,2}\cos\;\theta_{T}$, the energy of the auto-terms integration $S_{\mathrm{RCFT}}\left(\rho_{T};\theta_{T}\right)\ll E_{\max}$ due to incoherent integration or the fact that only part of the auto-terms energy is accumulated. Figure 2c,d depicts the coherent integration results of the Figure 2a obtained by the proposed RCFT method in the Radon domain, where only the auto-terms are accumulated into peaks, while the cross-terms and spurious peaks are almost completely suppressed. Although Figure 2c shows that there also exist the cross-terms on the RCFT plane, they are much smaller compared to the auto-terms. Unlike the traditional quadratic time-frequency distributions that usually exploit the smoothing, optimal kernel design or nonlinear filtering techniques to moderately tradeoff between the cross-terms and resolution, the proposed RCFT, on its own, can greatly “suppress” the cross-terms without any resolution loss. By “suppress”, we mean a relative suppression is achieved since the RCFT greatly strengthens the energy of the auto-terms instead of suppressing the cross-terms directly.

**Figure 3:**
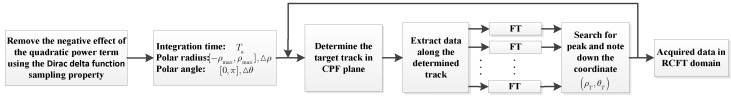
Detailed procedure of the proposed RCFT method.

From (12), interestingly, the proposed RCFT has similar operations as RCT and Hough-GHAF, and they all use the energy of the auto-terms along time-frequency trajectory in $t_{m}-f_{\tau_{m}^{2}}$ domain. The main difference is the coherent accumulation developed in the proposed RCFT. Therefore, the RCFT will definitely outperform the existing methods in complex environments via coherent integration operation. The detailed procedure of the proposed RCFT is shown in Figure 3.

The differences and advantages of RCFT compared with others approaches are briefly summarized as follows.

Remark 1: The RCFT employs the merits of both RCT and FT, and it not only has the same integration time as RCT but also works well as a useful tool for nonstationary signals.
Remark 2: The bilinear cubic phase function in (5) utilizes only one time correlation, which is viewed as a signal energy preservation because each additional one time correlation loses about 4 –5 dB in the SNR threshold [17]. In addition to that, the 2-D coherent integration realized in the proposed RCFT will further enhance the SNR. Therefore, the proposed RCFT algorithm provides a good performance, especially when the SNR is low, see simulation section.
Remark 3: the NUFFT speeds up the Fourier transform along the non-uniformly spaced lag-time axis, which is helpful for algorithm real-time realization.

### 3.2. Numerical Study of RCFT

**Figure 4:**
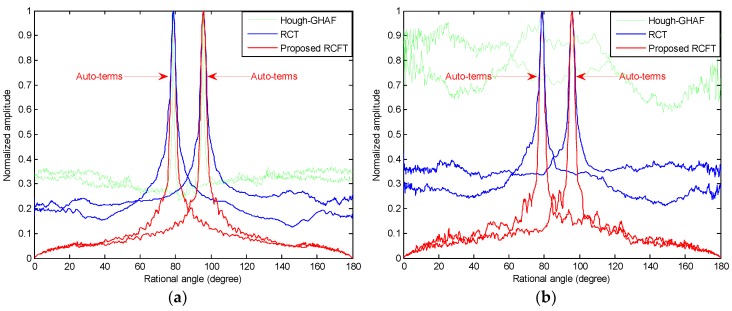
Comparison results of the three methods. (**a**) The slice obtained by the Hough-GHAF, RCT, and the proposed RCFT methods at SNR = 5 dB; (**b**) the slice obtained by the Hough-GHAF, RCT, and the proposed RCFT methods at SNR = −5 dB.

In this section, a simulation with the same parameters in Figure 2 is provided to demonstrate the performance comparisons with the RCT, and Hough-GHAF methods under different SNR conditions. Figure 4a,b depict the comparisons of RCT, Hough-GHAF, and the proposed RCFT with SNR being 5 dB and –5 dB, respectively. In Figure 4a, the cross-terms and spurious peaks are suppressed by all three approaches. However, both the RCT and Hough-GHAF are sensitive to noise. In Figure 4b, when SNR is low, say −5 dB, the spectrum of the Hough-GHAF is overwhelmed by the noise due to the non-coherent integration and multilinear function of fourth-order utilization. On the other hand, the RCT and the proposed RCFT are able to generate two distinct peaks at true locations. However, the sidelobes of RCFT are much lower than that of RCT due to the 2-D coherent integration, which indicates that the RCFT presents better cross-terms and noise suppression ability.

### 3.3. ISAR Imaging for Maneuvering Target Based on the Proposed RCFT Algorithm

In this section, an efficient ISAR imaging algorithm of targets with complex motions is developed, in which the FT in conventional RD algorithm is replaced by the proposed quasi-time-frequency analysis bilinear coherent RCFT. The main steps of the ISAR imaging algorithm are listed as follows.

Step 1: Perform the range compression and the translational motion compensation including envelope alignment and phase autofocus.
Step 2: Characterize the azimuth signal of a range cell after translational compensation as multi-component QFM signals $s_{r}\left(t_{m}\right)$, and perform NUFFT along the lag time variable to obtain CPF result $\mathrm{CPF}\left(t_{m},f_{\tau_{m}^{2}}\right)$.
Step 3: Apply the proposed RCFT to the $\mathrm{CPF}\left(t_{m},f_{\tau_{m}^{2}}\right)$ and obtain a three-dimensional data matrix ${\mathrm{RCFT}}_{cterms}\left(f_{t_{m}};\rho_{T};\theta_{T}\right)$ in the Doppler Centroid $f_{t_{m}}$-polar radius $\rho_{T}$-polar angle $\theta_{T}$ domain.
Step 4: Project the three-dimensional data matrix $\mathrm{RCFT}\left(f_{t_{m}};\rho_{T};\theta_{T}\right)$ onto the Doppler frequency axis along the polar radius $\rho_{T}$ and polar angle $\theta_{T}$, which is obtained by
  (16) $$\;s\left(f_{t_{m}\;}\right)=\arg\underset{\rho_{T};\theta_{T}}{\max}\left(|\mathrm{RCFT}\left(f_{t_{m}};\rho_{T};\theta_{T}\right)\right)$$

**Figure 5:**
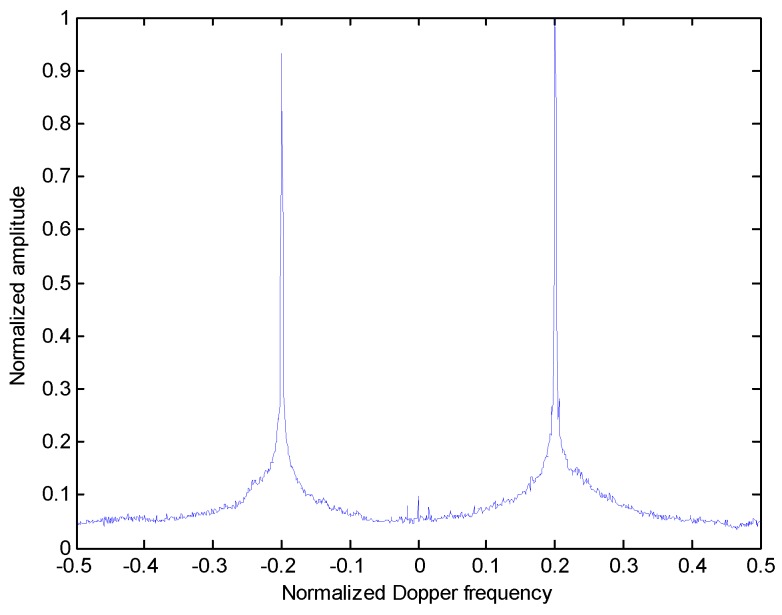
Maximization projection onto the Doppler frequency dimension.

It is a common knowledge that the Doppler frequency of each scatterer is proportional to its cross-range position in the target. Hence, the cross-range ISAR image of the target can be obtained by its projection onto the Doppler frequency axis. In Figure 5, the projection results of the RCFT shown in the Figure 2 along the Doppler frequency dimension is presented. It is seen that two distinct peaks appear along the Doppler frequency axis, which corresponds to two scatterer cross-range positions.

**Figure 6:**
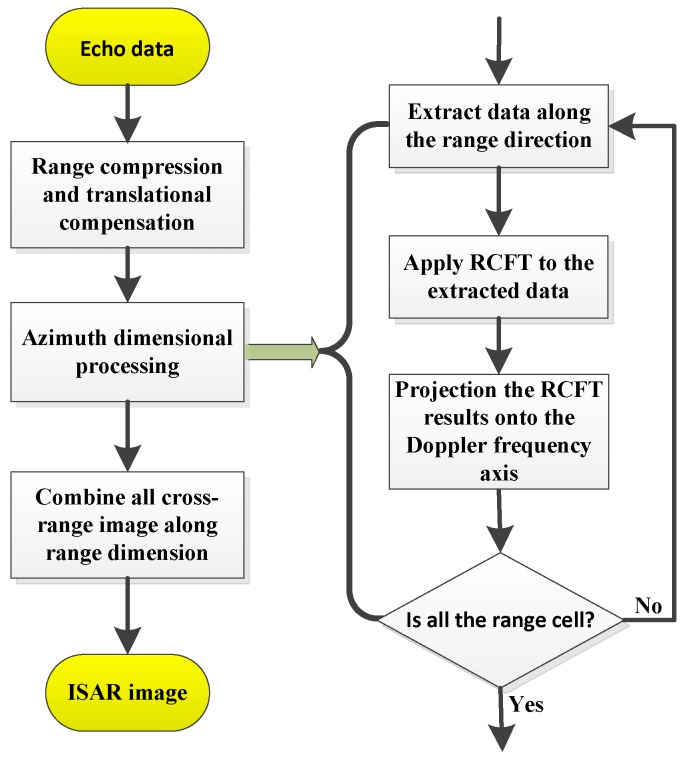
Flowchart of ISAR imaging based on the proposed RCFT.

Step 5: Set a proper extraction threshold or a filter to suppress the residual cross terms and noise in the Doppler centroid frequency dimension. In practice, the threshold is usually determined by subtracting −3~−4.5 dB from the maximal energy.
Step 6: Repeat the process of step 1–step 5 for all range cells, and the final high-resolution ISAR image is thus produced by regrouping all the range-Doppler frequency centroids. Since the proposed method does not require computations such as parameter estimation for each scatterer, it is computationally more efficient than the similar parameter estimation based algorithms. The flowchart of the proposed ISAR imaging algorithm is shown in Figure 6.

### 3.4. Components Computational Complexity Analysis

In this section, we analyze quantitatively the computational complexity of our proposed algorithm. For comparison purposes, the RCD method in [15] where the high-resolution ISAR image can be obtained based a new quasi-time-frequency transform named Lv’s distribution using the second-order phase model under a low SNR environment. Moreover, the cross-terms suppression in the RCD method is better achieved with no time-frequency resolution loss. On the other hand, to compare with the parameter estimation-based ISAR imaging method, the CIGCPF-CICPF algorithm [9] where it is recently proposed for maneuvering target ISAR imaging and parameter estimation with third-order motion model in low SNR condition.

In this comparison, for the illustration conveniences, assume that the range compression and translational motion compensation have been completed. The computational complexities of above-mentioned two methods and our proposed algorithm are quantitatively provided. In general, an *N*-point FFT or inverse FFT (IFFT) needs $5N\log_{2}\left(N\right)$ floating-point operations (FLOPs) and one-time complex multiplication needs 6N FLOPs. In what follows, $N_{r}$ and $N_{a}$ are respectively used to denote the number of range cells and the number of azimuth pulses, $K_{l}$ is the target scatterer number in the lth range cell, $N_{t_{m}}$ is the signal length $t_{m}$, and $N_{\tau_{m}}\;$is used to represent the length of the lag variable$\;\tau_{m}$.

For the RCD method, its implementation steps mainly include performing the quasi-time-frequency distribution (Lv’s distribution) to each range cell. Take a range cell processing procedure for example, the complex multiplication in constructing the symmetric instantaneous autocorrelation function matrix with computational complexity of $O\left(6N_{a}N_{\tau_{m}}\right)$, the FFT operation along the lag-time variable axis with computational cost of $O\left(5N_{a}N_{\tau_{m}}\log_{2}N_{\tau_{m}}\right)$, a keystone transform adopted to remove the coupling terms with complexity $O\left(2\left(2N_{ker}-1\right)N_{a}N_{\tau_{m}}\right)$, where $N_{ker}$ is the length of the interpolation operation kernel, the FFT operation along the scaling slow time variable with computational cost of $O\left(5N_{a}N_{\tau_{m}}\log_{2}N_{a}\right)$, and neglecting other relatively small computational complexity operation steps. Therefore, the total computational complexity of the RCD algorithm [15] is

(17) $$\;C_{\mathrm{RCD}\;}=O\left[N_{r}\left(6N_{a}N_{\tau_{m}}+5N_{a}N_{\tau_{m}}\log_{2}N_{\tau_{m}}+2\left(2N_{ker}-1\right)N_{a}N_{\tau_{m}}+5N_{a}N_{\tau_{m}}\log_{2}N_{a}\right)\right]\;$$

Compared with the parameter estimation-based method and our proposed method, which will be discussed later, the time-frequency analysis-based RCD method has a great advantage in terms of computational complexity. However, this method suffers from imaging performance degradation without considering the third-order phase effects.

For the parameter estimation-based CIGCPF-CICPF method where it needs to estimation each scatterer parameter, the computational load consists of the following steps. Take one scatterer estimation for example, to estimate the first- and third-order coefficient using the CIGCPF, the complex multiplication in constructing fourth-order multilinear GCPF function matrix with computational complexity of $O\left(18N_{a}N_{\tau_{m}}\right)$, the NUFFT operation along the lag-time variable axis with computational cost of $O\left(40N_{a}N_{\tau_{m}}\log_{2}N_{\tau_{m}}\right)$, one time compensation function multiplication with complexity $O\left(6N_{a}N_{\tau_{m}}\right)$, the FFT operation along the slow time variable with computational cost of $O\left(5N_{a}N_{\tau_{m}}\log_{2}N_{a}\right)$. Then one Dechirping operation is required, which needs one $N_{a}$-dimensional complex multiplication. Second, to obtain second-order coefficient using the CICPF, the computational complexity requirement is similar to the CIGCPF operation. Finally, one $N_{a}$-dimensional FFT is needed to estimate amplitude. Therefore, the total computational complexity of the CIGCPF-CICPF method [9] for maneuvering target imaging with third-order phase model is

(18) $$\;C_{\mathrm{CIGCPF}-\mathrm{CICPF}\;}=O\left[N_{r}\overset{N_{r}}{\underset{l=1}{\sum }}K_{l}\left(42N_{a}N_{\tau_{m}}+80N_{a}N_{\tau_{m}}\log_{2}N_{\tau_{m}}+10N_{\tau_{m}}N_{a}\log_{2}N_{a}+5N_{a}\log_{2}N_{a}\right)\right]$$

Similar to the RCD method, the proposed ISAR imaging algorithm is also based the quasi-time-frequency analysis named RCFT. According to the imaging steps and the flowchart of the proposed algorithm in Figure 6, the proposed algorithm implementation procedures mainly include applying the proposed RCFT to each range cell. Take a range cell processing procedure for example, in constructing bilinear CPF function matrix with computational complexity of $O\left(6N_{a}N_{\tau_{m}}\right)$ the NUFFT operation along the lag-time variable axis with computational cost of $O\left(40N_{a}N_{\tau_{m}}\log_{2}N_{\tau_{m}}\right)$, one time compensation function multiplication with complexity $O\left(6N_{a}N_{\tau_{m}}\right)$ and the auto-terms trajectory extraction in 2-D time-frequency $t_{m}-f_{\tau_{m}^{2}}$ domain and performing a FFT operation to the extracted data along slow-time variable with computational cost of $O\left(5N_{a}N_{\tau_{m}}\log_{2}N_{a}\right)$ with searching point number M. Therefore, the total computational cost of the proposed ISAR imaging method is about

(19) $$\;C_{\mathrm{Proposed}\;}=\;O\left[N_{r}\left(12N_{a}N_{\tau_{m}}+40N_{a}N_{\tau_{m}}\log_{2}N_{\tau_{m}}+5N_{\tau_{m}}MN_{a}\log_{2}N_{a}\right)\right]$$

According to the above analysis, the computational complexity of the proposed ISAR imaging is higher than that of the RCD method, but still much lower that of parameter estimation-based CIGCPF-CICPF approach. In conclusion, the proposed method may well achieve a trade-off between the computational complexity and the imaging performance, see performance analysis section. 

## 4. Simulation Results and Analysis

**Figure 7:**
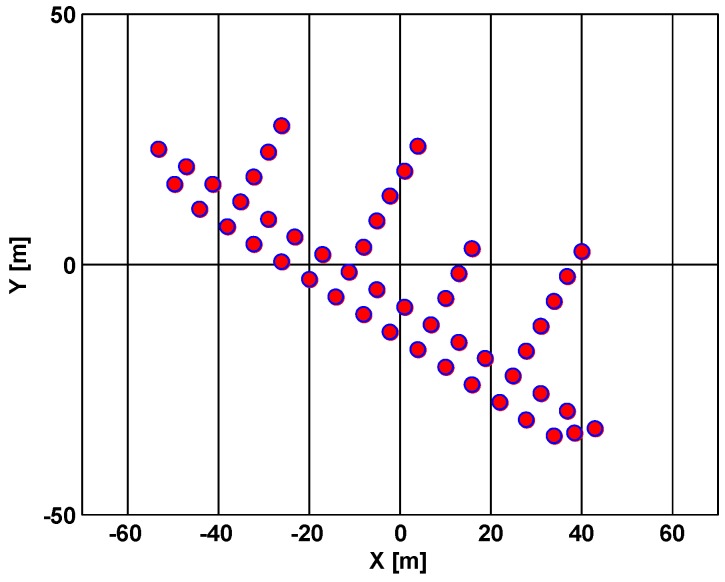
Vessel model of the ISAR imaging.

To confirm the validity of the proposed algorithm, simulation experiments are conducted now under two conditions of input SNR = 5 dB and SNR = −3 dB with the simulation parameters of the radar and the moving target listed in Table 1. The target scatterer model used in the simulation was a ship with 49 scatterers, shown in Figure 7.

**Figure 8:**
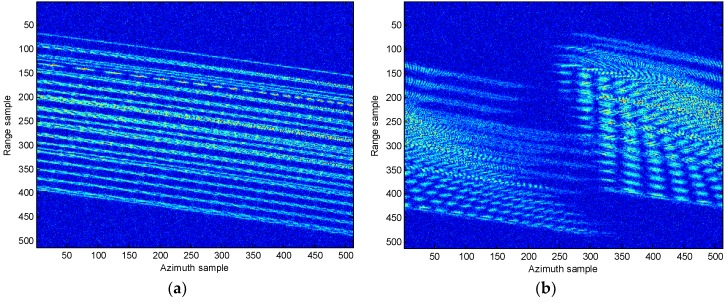
Range compression and imaging result without migration compensation. (**a**) Range compression; (**b**) RD imaging result.

**Figure 9:**
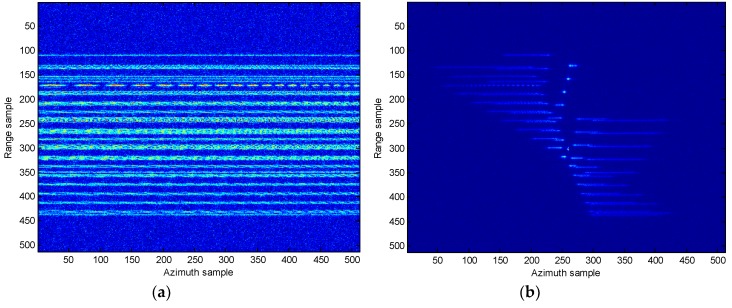
Range compression and imaging result with migration compensation. (**a**) Range compression; (**b**) RD imaging result.

In Figure 8, the range compression and imaging result using RD algorithm without the migration compensation are presented. It is clear that from Figure 8a the energy for the target disperses several range bins. In addition, the image produced by the RD algorithm is unclear and blurry. In Figure 9, the motion compensation including the translational compensation (envelope alignment and phase adjustment) and the migration through resolution cells (MTRCs) correction [9,23,24,25], is conducted. From Figure 9, it reveals that the range migration is fully rectified and the energy of the target is now concentrated into one range bin. It is therefore concluded that the migration compensation is effective must, and this step is always conducted first in the following experiments. In the following, the comparison results of different approaches are presented.

**Figure 10:**
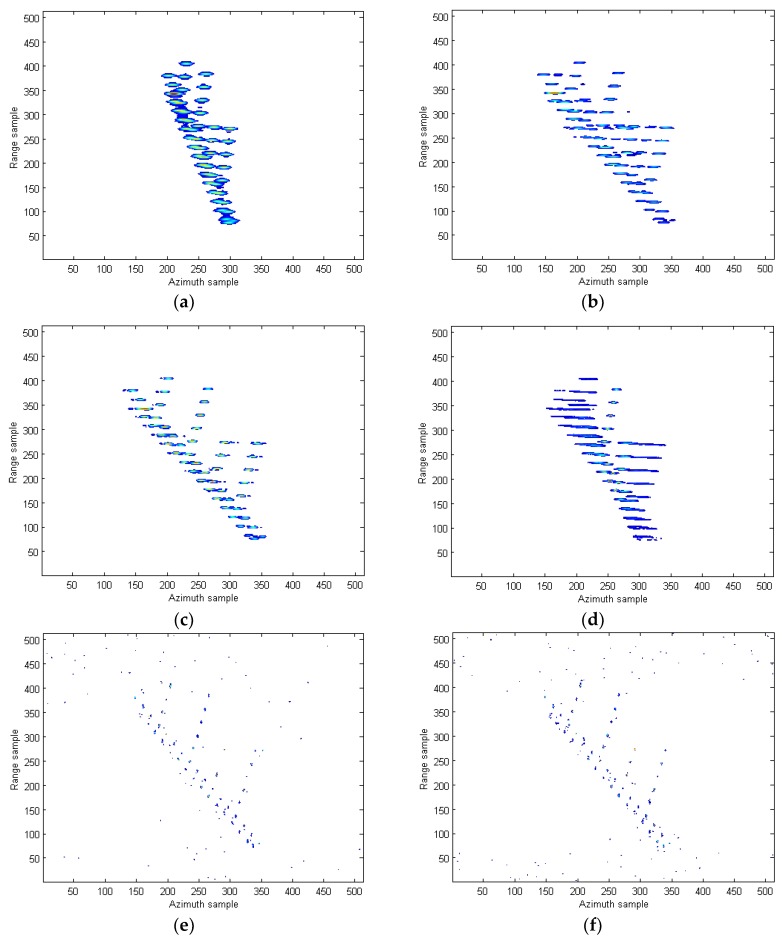

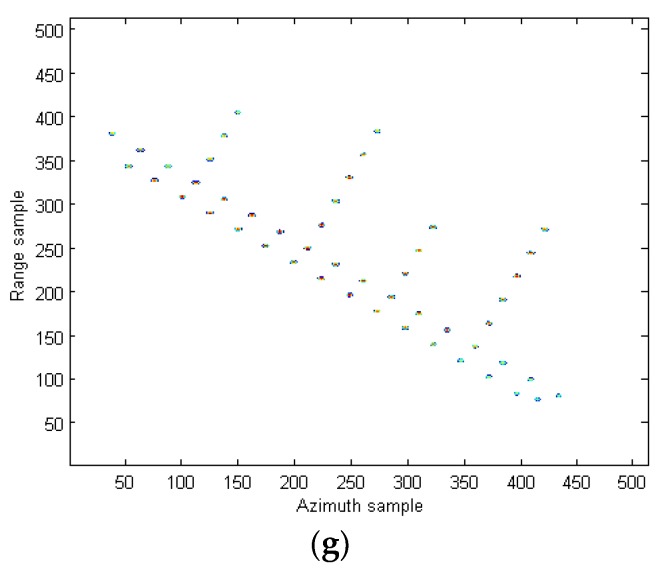
ISAR images of the simulated data under SNR = 5 dB. (**a**) STFT algorithm in [10]; (**b**) WVD algorithm in [12]; (**c**) SPWVD method in [13]; (**d**) RCD method in [15]; (**e**) IHAF-ICPF method in [7]; (**f**) CIGCPF-CICPF method in [9]; (**g**) our proposed method.

**Figure 11:**
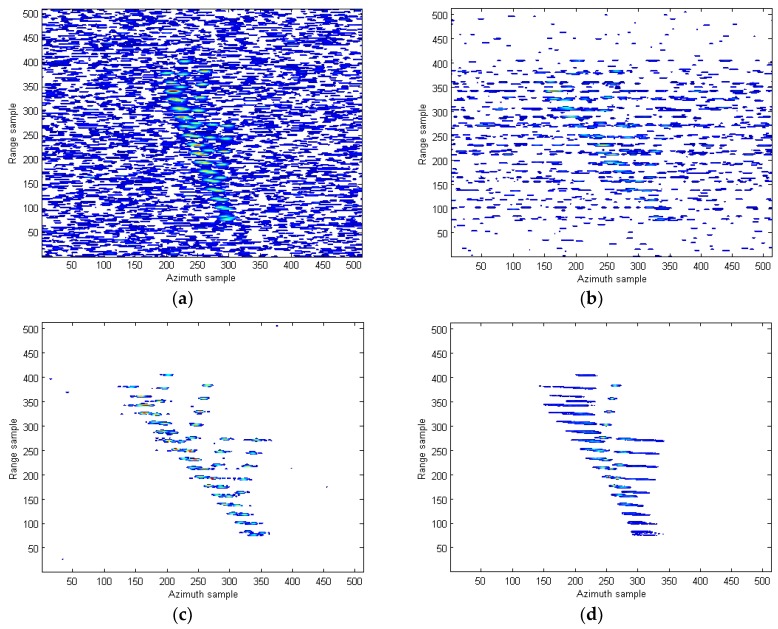

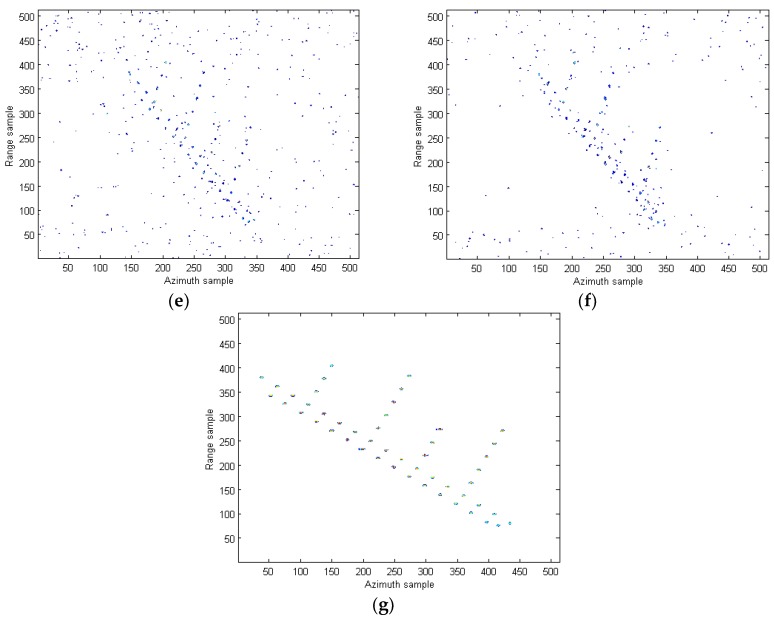
ISAR images of the simulated data under SNR = −3 dB. (**a**) STFT algorithm in [10]; (**b**) WVD algorithm in [12]; (**c**) SPWVD method in [13]; (**d**) RCD method in [15]; (**e**) IHAF-ICPF method in [7]; (**f**) CIGCPF-CICPF method in [9]; (**g**) our proposed method.

The 2-dimensional (2-D) ISAR image obtained by the RD algorithm under SNR = 5 dB is provided in Figure 9b. Since the Doppler frequency is time-varying, it is observed that the image is seriously blurred in the cross range. The images produced by STFT, WVD, and SPWVD are demonstrated in Figure 10a–c, respectively. Because of the cross-terms interference in WVD, the shape of ship is not visible. Suffering from low resolutions, the images from STFT and SPWVD are not focused well and each scatter spreads out in azimuth direction. In Figure 10d, the ISAR imaging result obtained by the RCD method in [15] is presented. As discussed earlier, since this method is only valid for LFM signal, the image obtained is seriously blurred in the cross range. To clearly demonstrate the advantages of the proposed approach over the existing methods, we also compare the proposed method with the parameter estimation-based algorithms. Figure 10e,f provide the 2-D SAR images obtained by the IHAF-ICPF method [7], CIGCPF-CICPF method [9] at SNR = 5 dB. It is observed that ship shape is generated by those two approaches, but they are not free of residual interference. On another note, parameter estimation-based algorithms are computationally extensive and suffer from model mismatch issue, which limit their usages. Figure 10g shows the high-quality ISAR image produced by the proposed quasi-time-frequency RCFT, where image is well focused in both range and azimuth directions, which agrees with our theoretical analysis. Figure 11 presents the ISAR images obtained by the aforementioned methods under SNR = −3 dB. Compared with other methods, under the low SNR condition, our proposed ISAR imaging algorithm still produces high quality image. This means most scatterers are relocated correctly with less scatterers loss and less artifact appearances as presented in Figure 11g. This superior performance obtained is all because the 2-D coherent integrations and bilinear features utilized in the proposed methods to suppress the cross-terms and spurious peaks, and at the same time resolution is not sacrificed.

To further evaluate the performance, the entropy of 2-D ISAR images is utilized to measure the image quality. It is well known that a better quality image indicates a smaller entropy [26,27,28]. The entropy for an image g(m,n) is

(20) $$\;I=\overset{M-1}{\underset{m=0\;}{\sum }}\overset{N-1}{\underset{n=0}{\sum }}\frac{{\left|g\left(m,n\right)\right|}^{2}}{S}ln\frac{S}{{\left|g\left(m,n\right)\right|}^{2}}$$

where $S=\sum_{m=0}^{M-1}\sum_{n=0}^{N-1}{\left|g\left(m,n\right)\right|}^{2}$. Entropies obtained by these methods are listed in Table 2, where the smallest values are obtained by the proposed imaging algorithm. This is in the agreement with the conclusions obtained in Figure 10 and Figure 11 since the proposed method produces the clearest ship model free of artifacts, and also demonstrate the effectiveness of the proposed ISAR imaging algorithm under low SNR condition.

**Figure 12:**
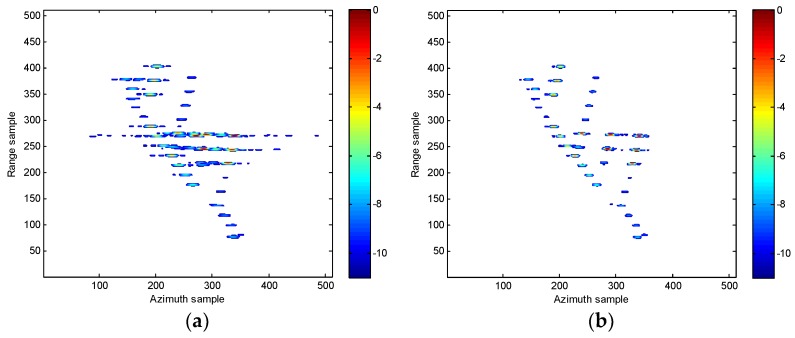

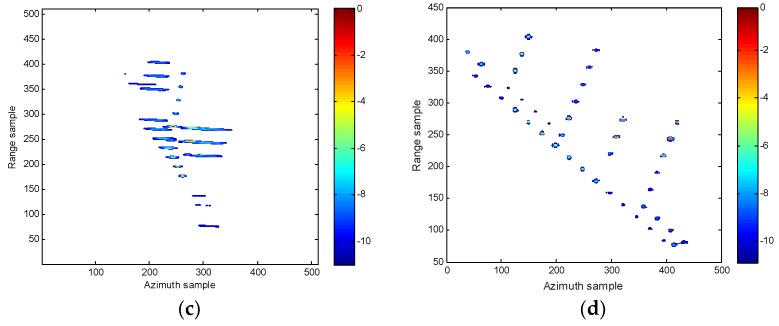
ISAR images of the simulated data under SNR = 5 dB in the case of uneven strengths of scatterers. (**a**) WVD algorithm in [12]. (**b**) SPWVD method in [13]. (**c**) RCD method in [15]. (**d**) Our proposed method.

In order to imitate the measured data environment, we have added a simulation analysis. In this simulation, the amplitudes of scatterers vary randomly from 0.1 to 1, and the corresponding results are provided in Figure 12 It clearly shows that the few weak scatterers are not prominent anymore, but most scatterers are still present to form the shape of the target. In fact, all the nonlinear time-frequency analysis approaches are actually challenged by the issue of weak scatterers.

The implementation times of different methods are provided in Table 3, which are obtained using a computer with double core CPU 3.4 GHz and memory of 8 G. From the table, the running times of time-frequency analysis (STFT, WVD, and SPWVD methods)-based approaches are lowest and the parameter estimation-based ISAR imaging approaches of IHAF-ICPF method and CIGCPF-CICPF are highly time consuming. The time cost of proposed RCFT is higher than the time-frequency analysis based approaches, but much lower than the parameter estimation-based approaches. Taking both the performance and computation into consideration, to conclude, the proposed method well trades off them in producing clear image of the maneuvering target at low SNR environment.

## 5. Conclusions

In this paper, the RCFT method is first proposed for the analysis of multiple QFM signals. Because of 2-D coherent integration realization and the bilinear function feature, the better cross-term interference suppression is achieved at no loss of time-frequency resolution, and also higher signal processing gain is obtained. The RCFT is computationally efficient since no expensive three-dimensional (3-D) parameter search is required. After that, the RCFT is applied to the ISAR imaging problem for a maneuvering target in producing the clear image. Numerical results demonstrate that the proposed method outperforms existing ISAR imaging algorithms in terms of both visual inspections and objective performance measures.

## Figures and Tables

**Table 1:** Radar parameters and target moving model.

Parameters Name	Value
Carrier frequency	10 GHz
Transmit bandwidth	200 MHz
Pulse repetition	300 Hz
Wave length	0.03 m
Range sample frequency	300 MHz
Effective echo pluses	512
Translational coefficients velocity	20 m/s
Translational coefficients acceleration	2 m/s^2^
Translational coefficients acceleration rate	2 m/s^3^
Effective rotational motion angular velocity	0.018 rad/s
Effective rotational motion acceleration	0.008 rad/s^2^
Effective rotational motion acceleration rate	0.002 rad/s^3^

**Table 2:** Entropies of ISAR Images in Figure 10 and Figure 11.

Methods	SNR = 5 dB		SNR = −3 dB	
	Figure	Entropy	Figure	Entropy
STFT method in [10]	Figure 10a	9.905	Figure 11a	11.623
WVD method in [12]	Figure 10b	9.170	Figure 11b	11.341
SPWVD method in [13]	Figure 10c	7.679	Figure 11c	9.208
RCD method in [15]	Figure 10d	7.418	Figure 11d	7.637
IHAF-ICPF method in [7]	Figure 10e	4.870	Figure 11e	5.619
CIGCPF-CICPF method in [9]	Figure 10f	4.513	Figure 11f	5.037
Our proposed method	Figure 10g	4.291	Figure 11g	4.372

**Table 3:** Computational complexity comparisons.

Methods	Runtime
RD algorithm	0.50 m
STFT method	1.24 m
WVD method	1.56 m
SPWVD method	2.73 m
RCD method	5.04 m
IHAF-ICPF method	56.23 m
CIGCPF-CICPF method	41.78 m
Our proposed method	8.35 m
